# *Bacillus Cereus* bacteremia complicated by brain abscess in a severely immunocompromised patient: Addressing importance of early recognition and challenges in diagnosis

**DOI:** 10.1016/j.idcr.2022.e01525

**Published:** 2022-06-06

**Authors:** David Schoenfeld, Dasom Lee, John A. Arrington, John Greene, Olga Klinkova

**Affiliations:** a Department of Internal Medicine, University of South Florida, 17 Davis Blvd., Suite 308, Tampa, FL 33606, USA; b Department of Radiology, Moffitt Cancer Center, 12902 USF Magnolia Drive, Tampa, FL 33612, USA; c Infectious Disease Division, Moffitt Cancer Center, 12902 USF Magnolia Drive, Tampa, FL 33612, USA

**Keywords:** CNS, Central Nervous System, AML, Acute Myeloid Leukemia, B. cereus, Bacillus cereus, rRNA, ribosomal ribonucleic acid, PCR, polymerase chain reaction, IV, intravenous, CT, computer tomography, CSF, cerebrospinal fluid, MRI, magnetic resonance imaging, *Bacillus cereus*, Brain abscess, Immunocompromised, 16S rRNA PCR sequencing, CNS infection, Combination antibiotic therapy

## Abstract

*Bacillus cereus* (*B. cereus*) is a known cause of a food poisoning in the general population. However, it can cause life-threatening sepsis and shock in severely immunocompromised patients with hematologic malignancies, which frequently lead to central nervous system (CNS) infections associated with high mortality and morbidity. In this case report, we describe a patient with a newly diagnosed acute myeloid leukemia that underwent induction chemotherapy and developed *B. cereus* infection that was associated with septic shock and brain abscesses. Definitive diagnosis of multiple brain abscesses was not manifested with routine microbiological investigation but required the use of 16S ribosomal (rRNA) gene polymerase chain reaction (PCR) sequencing of the resected brain lesion. The patient was eventually treated with 8-week course of intravenous vancomycin and high-dose ciprofloxacin which led to a full recovery. This report highlights the significant risk posed by *B. cereus* infection in neutropenic patients, the use of 16S rRNA PCR sequencing test for definitive diagnosis and use of combination therapy for successful treatment of *B. Cereus* CNS infection.

## Introduction

*Bacillus cereus (B. cereus)* is a gram-positive, facultatively anaerobic, spore forming bacterium that can be easily found in the environment [1]. It is commonly associated with self-limiting food poisoning in healthy individuals as it abundantly produces preformed toxins that cause acute gastroenteritis. Spores of *B. cereus* as well as other *Bacillus* species are also ubiquitous in the hospital environment and can be the cause of pseudonosocomial infections [2]. Pseudo-outbreaks of *Bacillus* have been traced to contaminated ethyl alcohol, hospital linen, gloves, blood culture media and hospital buildings [3], [4], [5], [6]. Therefore, due to its ubiquity in the nosocomial setting, *Bacillus* species is often considered a contaminant when resulted in blood cultures in the general population. However, immunocompromised patients, especially those with hematologic malignancies, are at high risk for developing severe *B. cereus* infections. It can present in various forms, ranging from bacteremia to life-threatening infections such as septic shock and central nervous system (CNS) infections [7], [8], [9], [10], [11].

In this study, we describe a patient who developed *B. cereus* bacteremia with sepsis, which was later complicated by the formation of brain abscesses. Different microbiologic methods were used to confirm the etiology of the brain lesions, including broad range 16 S ribosomal ribonucleic acid (rRNA) gene polymerase chain reaction (PCR). Our patient was successfully treated with a prolonged antibiotic course and achieved a full recovery.

## Case presentation

**Fig. 1:**
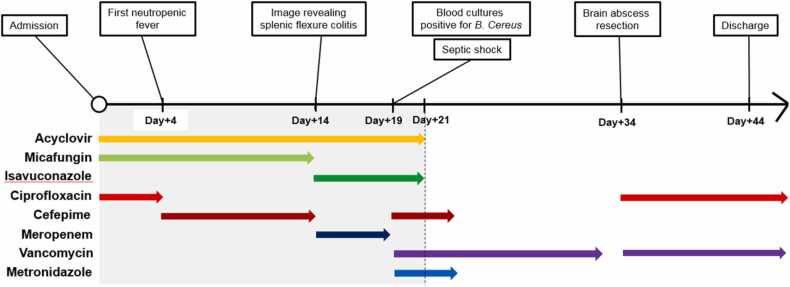
Hospital course of the patient from admission (day 0) to the day of discharge (day+44). Lymphocytopenia (defined as count <1000 cells/microL) and severe neutropenia (defined as count <500 cells/microL) were present from day 0 to day+ 21 and colored in grey. Significant clinical events and sequential antibiotic treatment were indicated above and below timeline, respectively.

A 55-year-old African American female patient first presented to the outside hospital emergency room for chief complaint of fatigue and unprovoked ecchymosis. Basic workup demonstrated a new finding of neutropenia with absolute neutrophil count (ANC) of 0.26 k/uL and anemia with hemoglobin of 9.1 g/dL. Prophylaxis with oral acyclovir, intravenous (IV) micafungin and oral ciprofloxacin was initiated. Bone marrow biopsy was performed and revealed acute myeloid leukemia (AML), specifically, acute myelomonocytic leukemia type. The patient was admitted to our institution for the initiation of induction chemotherapy regimen called “7 + 3 +GO” (cytarabine on days1–7, daunorubicin on days1–3, gemtuzumab ozogamicin on day 1). Peripherally inserted central venous catheter was placed on admission (Fig. 1).

On hospital day 4, our patient developed neutropenic fever and oral ciprofloxacin prophylaxis was escalated to IV cefepime. She remained on prophylactic oral acyclovir and IV micafungin. Blood and urine cultures were negative for infection, and chest and sinuses computer tomography (CT) were negative for acute pathology. Despite the negative microbiologic work-up and imaging data, the patient continued to have persistent neutropenic fever. On hospital day 14, repeat CT imaging of the chest revealed findings of splenic flexure colitis though blood cultures continued to be negative. Given persistent neutropenic fever with evidence of colitis, cefepime was changed to IV meropenem. This led to the resolution of the fever and clinical improvement. She continued oral acyclovir prophylaxis and IV micafungin was transitioned to oral isavuconazole in view of prolonged neutropenia.

On hospital day 19, our patient developed recurrent neutropenic fever with maximum temperature of 104.5 F with hypotension, new onset confusion and lethargy without focal neurologic deficits. Investigation including brain CT and electroencephalography did not favor acute neurological process. The patient required transfer to medical intensive care unit for the management of septic shock. She continued on IV meropenem, and IV vancomycin was added for additional gram-positive coverage; pressors were initiated for hypotension. Subsequently, two out of two blood cultures resulted positive for *B. cereus* in both aerobic and anaerobic bottles on hospital day 19. With this available culture data, meropenem was de-escalated back to IV cefepime and IV metronidazole. She remained on IV vancomycin for the treatment of *B. cereus* bacteremia. Repeat blood cultures that were obtained in less than 2 days were negative. The origin of *Bacillus* bacteremia was unclear but gut translocation in setting of profound neutropenia versus catheter-associated infection were considered. Due to clinical improvement and rapid clearance of bacteremia, the peripherally inserted central venous catheter was not removed.

Despite clearance of the bloodstream infection, resolution of fever and neutrophil recovery, the patient continued to be encephalopathic with on-going confusion and intermittent lethargy. On hospital day 21, magnetic resonance imaging (MRI) of the brain was performed and demonstrated multifocal enhancing lesions concerning for leukemic involvement, formation of abscess or ischemic changes. Head and neck CT angiography and bilateral carotid artery ultrasound were non-revealing and transthoracic echocardiogram with bubble study was negative for valvular vegetations. Cerebrospinal fluid (CSF) analysis was obtained via lumbar puncture and was significant for elevated number of white blood cells at 98 (normal range 0–5) with neutrophil predominance at 66% and red blood cells of 45 (normal range 0). CSF protein was elevated at 97 mg/dL (normal range 40–70 mg/dL), glucose was elevated at 72 mg/dL (normal range 15–45 mg/dL), cytology was significant for cellular atypia concerning for blasts. CSF microbiologic studies such as gram stain, bacterial, fungal cultures and multiplex meningitis/encephalitis panel by PCR were all negative. As the patient’s ANC rose above 500 cells/uL on hospital day 21, prophylaxis with isavuconazole and acyclovir were discontinued. On hospital day 24, IV cefepime and IV metronidazole were discontinued after completion of 10 day-course of treatment for suspected colitis and IV vancomycin was continued for the total of 14 days of therapy.

**Fig. 2:**
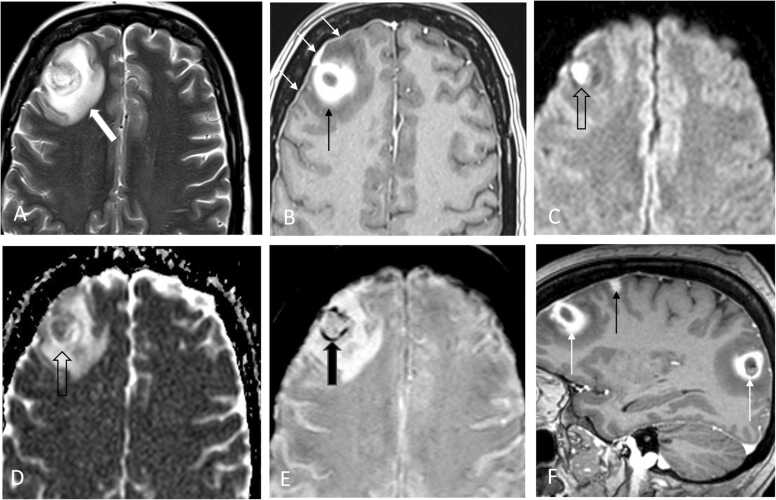
MRI imaging of brain on hospital day 34. Axial T2W (A), contrast enhanced T1W (B), B1000 DWI (C), ADC diffusion map (D), and SWI (E) MRI images of the brain demonstrate a ring enhancing lesion right frontal cortex (black arrow) with associated dural thickening and enhancement over the right frontal cortex (white arrows) and surrounding vasogenic edema (white block arrow). The lesion demonstrated restricted diffusion seen as increased signal on DWI and decreased signal on ADC map images (open block arrow) as well as evidence of petechial hemorrhage seen on SWI (black block arrow). Sagittal contrast enhanced T1W (F) MRI image of the brain demonstrates ring enhancing lesions frontal and temporal lobe (white arrows) and smaller solid enhancing lesion frontal cortex (black arrow).

Even though the patient’s mentation significantly improved, she continued to experience intermittent episodes of confusion. On hospital day 34, repeat brain MRI was obtained and was significant for interval progression of brain lesions (Fig. 2). As prior, differential diagnosis included abscess formation or leukemic involvement. At that point, blood cultures were repeated, and the patient was empirically restarted on IV vancomycin in view of recent *B. cereus* bacteremia. Neurosurgery service was consulted for brain biopsy to establish the diagnosis of the brain lesions. On hospital day 35, the patient underwent image guided right frontal craniotomy for resection of a right frontal superficial brain mass. Intraoperatively, gross purulence was observed upon entering the mass. Pathology examination was suggestive of a treated bacterial abscess even though no infectious organism was identified on stains.

On hospital day 36, in addition to IV vancomycin, IV ciprofloxacin 400 mg every 8 h was started for additive treatment of possible *B. cereus* infection while awaiting tissue cultures from recent brain biopsy. Blood cultures continued to be negative. Intraoperative bacterial tissue cultures were finalized as negative at three days. Broad range 16 S rRNA gene PCR sequencing was requested on the brain tissue sample to help establish a microbiologic diagnosis. The patient’s mental status continued to improve on combination IV ciprofloxacin and IV vancomycin over the hospital course. On hospital day 43, the patient was discharged with plan to complete eight weeks of combination therapy with IV vancomycin and oral ciprofloxacin 750 mg twice a day.

Two weeks after brain biopsy was completed, bacterial PCR testing returned positive for *B. cereus* confirming the diagnosis. Six weeks after discharge, the patient followed up in our outpatient infectious disease clinic. The patient was doing clinically well with the complete resolution of her prior neurologic symptoms. Follow-up MRI of brain at the completion of the treatment demonstrated residual changes and no new areas of enhancement. The patient’s dual antibiotic therapy was stopped at eight weeks. At the time of this manuscript submission, the patient remained asymptomatic and was in remission from her underlying hematologic malignancy.

## Discussion

Here, we describe a case of *B. cereus* bacteremia leading to septic shock and CNS infection in a patient with AML after induction chemotherapy. In this study, we highlight the importance of recognition of this particular infection in severely immunocompromised patients as well as the use of next generation 16 S rRNA sequencing for definitive diagnosis of *B. cereus* brain abscess.

Encephalitis, meningitis, and brain abscess caused by *B. cereus* are often found in post-intracranial surgery/device placement patients, neonates, or patients with severe neutropenia, often in the setting of hematologic malignancy [7], [8], [9], [10], [11], [12]. Specifically, the patients with AML undergoing induction chemotherapy are at high risk of developing *B. cereus* CNS infection once they experience ≥ 10 days of neutropenia and the infection can lead to death within days with the mortality rate of 79% [9]. Similarly, our patient received 7-day course of induction chemotherapy for newly diagnosed AML and developed septic shock with acute encephalopathy secondary to *B. cereus* bacteremia on hospitalization day 19. Definitive diagnosis of *B. cereus* CNS infection was challenging, because acute encephalopathy was initially thought to be attributed to septic shock. In addition, MRI of the brain obtained due to persistent encephalopathy after development of septic shock had a broad differential including extramedullary leukemia, infection as well as cerebrovascular disease. Though MRI of the brain on admission was normal, leukemic involvement could not be ruled out given the fact that cells with blast appearance were seen in the CSF analysis. CSF cultures as well as the tissue culture of the resected brain lesion on hospitalization day 34 were finalized as negative as well. Low sensitivity of both cerebrospinal fluid and brain tissue cultures can be explained by concomitant antibiotic administration that resulted in a loss of pathogen detection. These all together make this case challenging for definitive diagnosis and, potentially, could have resulted in significant complications [9], [10].

We were only able to reach definitive microbiologic diagnosis on the brain lesions by using next generation 16 S rRNA sequencing method. This technique utilizes the genus and species-specific variations found in the otherwise highly conserved 16 S rRNA, responsible for binding of the 30 S ribosomal subunit to the Shine-Delgarno sequence on messenger RNA to determine what bacterial species are present in a sample [13]. Strains having < 3% difference between their 16 S rRNA genes were considered the same species [14]. *B. anthracis, B. cereus, B. wiedmannii,* and *B. thuringensis* have < 1% difference in 16 S sequences and can be considered as one species [15], [16]. However, as shown in our case, direct detection of bacterial DNA performed at the University of Washington was able to further sub-speciate *B. Cereus,* which correlated with the result of blood cultures drawn to identify *B. cereus* bacteremia in our patient.

Treatment of *B. cereus* bacteremia consists of early administration of IV vancomycin, which is considered a preferred drug, or an alternative agent that has been shown to be effective based on the prior research or sensitivity testing [17], [18]. In vitro, *B. cereus* isolates demonstrated susceptibility to vancomycin, carbapenems, ciprofloxacin, selected aminoglycosides and chloramphenicol [17], [18]. Typical duration of therapy for uncomplicated blood stream infections is 7–14 days depending on the clearance of bacteremia, presence of the intravascular catheter and clinical response. There are no standard recommendations for the treatment *B. cereus* CNS infections, however, combination therapy of IV vancomycin with an alternative agent appears to be preferred and recommended per prior studies [7], [8], [10], [11]. Successful resolution was previously demonstrated with treatment regimen of vancomycin and meropenem [11] or vancomycin and ciprofloxacin [8]. For our case, the antibiotic sensitivity on the blood cultures positive for *B. cereus* was not evaluated per the institutional protocol. The drainage from the resected brain lesion was cultured, but there was no growth for identification or antibiotic sensitivity. The choice of vancomycin in combination of high dose ciprofloxacin was selected for several considerations. This combination of antibiotics has a narrower antibiotic coverage than vancomycin plus meropenem combination. In addition, ciprofloxacin was shown to effectively treat CNS infections especially given a good blood-brain barrier penetration in an aggressive dosing strategy [19], [20]. Eight-week course of combination therapy with IV vancomycin and high-dose ciprofloxacin resulted in a complete resolution of the brain abscesses in our case.

In summary, our patient exemplifies a unique case of *B. cereus* brain abscess following bacteremia. This case highlights the significant risk posed by *B. cereus* infection in neutropenic patients, and particularly neurologic involvement in the setting of concurrent hematologic malignancy. Rapid recognition and treatment of suspected *B. cereus* is critical given high patient mortality in neurologic infection. Lastly, genetic sequencing modalities such as 16 S rRNA sequencing offer a highly sensitive tool for definitive diagnosis in order to assist with proper antibiotic management and duration of antimicrobial therapy.

## CRediT authorship contribution statement

**David Schoenfeld, Dasom Lee, Olga Klinkova**: Wrote the first and final draft of the manuscript. **John A. Arrington**: Provided radiologic figures. **John Greene**: Wrote the final draft of the manuscript.

## Declaration of Interest

None.
